# Harvesting *Aurantiochytrium* sp. SW1 via Flocculation Using Chitosan: Effects of Flocculation Parameters on Flocculation Efficiency and Zeta Potential

**DOI:** 10.3390/md21040251

**Published:** 2023-04-19

**Authors:** Nadzirul Zamri, Nurul Nabila Suleiman, Norsyaqira Mohd Johar, Nur Syahidah Mohd Noor, Wei Lun Ang, Nazlina Haiza Mohd Yasin, Yusuf Nazir, Aidil Abdul Hamid

**Affiliations:** 1 Department of Biological Sciences & Biotechnology, Faculty of Science and Technology, Universiti Kebangsaan Malaysia, Bangi 43600, Malaysia; 2 Department of Chemical and Process Engineering, Faculty of Engineering & Built Environment, Universiti Kebangsaan Malaysia, Bangi 43600, Malaysia; 3 Department of Food Sciences, Faculty of Science and Technology, Universiti Kebangsaan Malaysia, Bangi 43600, Malaysia; 4 Innovation Centre for Confectionery Technology (MANIS), Faculty of Science and Technology, Universiti Kebangsaan Malaysia, Bangi 43600, Malaysia

**Keywords:** flocculation, chitosan, biomass harvesting, thraustochytrids, zeta potential

## Abstract

The use of chitosan as a flocculant has become a topic of interest over the years due to its positively charged polymer and biodegradable and non-toxic properties. However, most studies only focus on microalgae and wastewater treatment. This study provides crucial insight into the potential of using chitosan as an organic flocculant to harvest lipids and docosahexaenoic acid (DHA-rich *Aurantiochytrium* sp. SW1 cells by examining the correlation of flocculation parameters (chitosan concentration, molecular weight, medium pH, culture age, and cell density) toward the flocculation efficiency and zeta potential of the cells. A strong correlation between the pH and harvesting efficiency was observed as the pH increased from 3, with the optimal flocculation efficiency of >95% achieved at a chitosan concentration of 0.5 g/L at pH 6 where the zeta potential was almost zero (3.26 mV). The culture age and chitosan molecular weight have no effect on the flocculation efficiency but increasing the cell density decreases the flocculation efficiency. This is the first study to reveal the potential of chitosan to be used as a harvesting alternative for thraustochytrid cells.

## 1. Introduction

Long-chain ω-3 polyunsaturated fatty acids (PUFAs) such as docosahexaenoic acid (DHA, C22:6n3) and eicosapentaenoic acid (EPA, C20:5n3) play various important roles in human physiology, especially for the development of neural and retinal tissues [1]. However, humans cannot synthesize these fatty acids due to insufficient levels of elongates and delta-6-desaturases [2]. Although the conversion of alpha-linolenic acid into omega-3 fatty acids such as docosahexaenoic acid occurs in humans, it happens at a very slow rate and thus it must be acquired from dietary sources [3]. Hence, they are considered essential fatty acids. At present, oil extracted from marine fatty fish such as salmon and tuna is the major source of DHA [4]. However, the expanding demand for DHA worldwide is now placing pressure on both fisheries and the fish oil supply, and it is expected to be unable to meet the global market demand soon [5]. There are also possible health risks associated with the consumption of fish oils, such as food poisoning and allergies, particularly when there are issues of seawater contamination or toxicity and outbreaks of fish diseases [6]. Considering the disadvantages of fish-oil-based DHA and in order to achieve better commercial gains, efforts have been made to find alternative sustainable sources for omega-3 fatty acid production.

Thraustochytrids such as *Aurantiochytrium*, *Schizochytrium*, and *Thraustochytrium* are marine heterotrophic protists that have shown significant potential to be used for the commercial production of DHA as they are capable of producing up to 35–55% DHA from the total fatty acids (TFAs) as well as other interesting compounds such as carotenoids and squalene [7,8]. The production of DHA from thraustochytrids commonly involves upstream and downstream processing. Upstream processing such as strain development, as well as the development of fermentation processes that induce the cells to produce high biomass and lipid production, has been extensively studied and developed to date [9,10,11,12]. However, less focus has been placed on the development of downstream processing, especially the harvesting and dewatering of the lipid biomass, even though it is one of the major hurdles in lipids and DHA production associated with these microorganisms.

The main issues during downstream processing concerning cell recovery are harvesting efficiency and cost. Various approaches have been utilized in the recovery of cells of microalgae (photosynthetic Stramenopiles) such as centrifugation, filtration, gravity sedimentation, flotation, and flocculation [13]. None of these approaches combine techno-economic feasibility factors such as harvesting effectiveness costs and energy efficiency. It is reported that the cost of harvesting cell biomass is up to 20–30% of the cost of the entire downstream processes [14,15], primarily due to the dewatering process, which involves bulk water removal using a conventional technique such as centrifugation and filtration to separate biomass from the medium. Currently, the majority of thraustochytrids cells are harvested using the centrifugation method. Although effective for harvesting thraustochytrids cells, only up to 80% of the cells can be harvested, and it is also energy and cost-intensive [16], especially if large-scale cultures are used. A study conducted by Wong et al. [17] on *Aurantiochytrium mangrovei* MP2 cultures showed that after centrifugation, in addition to the pellet, a thick fraction consisting of vegetative cells containing lipid droplets was also observed in the top fraction of the supernatant. In the same study, the DHA concentrations in the top fraction were significantly higher compared to those in the bottom layer (up to 54.16%). Kim et al. [18] also reported the same phenomenon where centrifugation of *Aurantiochytrium* sp. KRS101 cultures at 9000× *g* for 30 min results in two similar fractions, which reduce cell recovery by 12.8–15.4%. Additionally, a similar observation was also reported by Patel et al. [19] where significant amounts of *Schizochytrium limacinum* cells (the harvesting efficiency was not indicated) were not able to be recovered after centrifugation at 8000 rpm (7881× *g*). Other methods, such as filtration, are ineffective as the filter paper’s pores are often clogged, which slows down the harvesting process [20]. Therefore, it is essential to explore potential approaches that are cost-effective and efficient for the harvesting process.

Alternatively, the flocculation method has been identified as one of the most low-cost and effective harvesting methods for harvesting thraustochytrids [18,20] and microalgal cells. The application of flocculation occurs through the neutralization of the surface charge of the cells resulting in flocs formation; hence, facilitating sedimentation via gravity has attracted researchers’ attention due to its simplicity and efficiency. Despite efficiencies, most reported flocculation studies use inorganic flocculants such as aluminum sulphate [AL_2_(SO_4_)_3_] and ferric chloride (FeCl_3_), which could potentially contaminate the final extracted lipid product [21]. Therefore, organic-based flocculants, such as chitosan, are preferred due to their non-toxic and biodegradable properties [22,23].

Chitosan is a cationic polyelectrolyte that can be obtained from chitin in fungi and exoskeletons of aquatic life and insects. Many studies have reported chitosan as an effective flocculant for harvesting microalgae cells such as *Chlorella* sp. and *Nannochloropsis* sp. [22,24,25,26]. However, to the best of the author’s knowledge, no study has been conducted using chitosan to harvest the thraustochytrids cells. Flocculation efficiency is reported to be dependent on the cell species [27]. This is because different cell species produce different amounts of biomass with different lipid content. For example, thraustochytrids produce lipids over 50% (g/g biomass), rendering the cells to be more challenging to sediment with higher biomass concentrations of up to 20 g/L compared to photosynthetic microalgae cultures where low biomass concentrations (<5 mg/L) are involved. Furthermore, different species also have different surface charge values, and the zeta potential of different cells could be differently affected by various parameters such as the cell density, type of medium used, age of the culture, and culture pH, which will affect the charge neutralization process during flocculation.

Flocculation has the potential to overcome the problems mentioned above by being an alternative harvesting method or easing the burdens of the dewatering step, which will further improve subsequent harvesting methods, such as centrifugation or filtration. A two-step harvesting procedure has been reported to be economically better than the single-step harvesting procedure where it can save costs and increase biomass recovery [28]. For example, flocculation will lower the cost of the subsequent dewatering step by preconcentrating the biomass from the medium and maximizing water removal before the final dewatering by centrifugation can be performed using the concentrated biomass [29]. This was also recommended by Kim et al. [18] where coagulation of thraustochytrid cells prior to dynamic filtration would result in the reduction of dewatering operation time from 180 min to 90 min. Therefore, this work represents the first intensive report focusing on the application of chitosan in harvesting lipids and DHA-rich thraustochytrids. In this study, the effects of flocculation parameters such as the flocculant concentration, flocculant molecular weight, medium pH, culture age, and cell density on the flocculation efficiency of *Aurantiochytrium* sp. SW1, a Malaysian thraustochytrid capable of producing between 50 and 60% lipids (g/g biomass) containing up to 50% DHA [30], was investigated. Furthermore, the correlation between the zeta potential, which represents the degree of charge neutralization between chitosan and cells’ flocculation efficiency, was evaluated.

## 2. Results and Discussion

### 2.1. Effect of Chitosan Concentration

**Figure 1:**
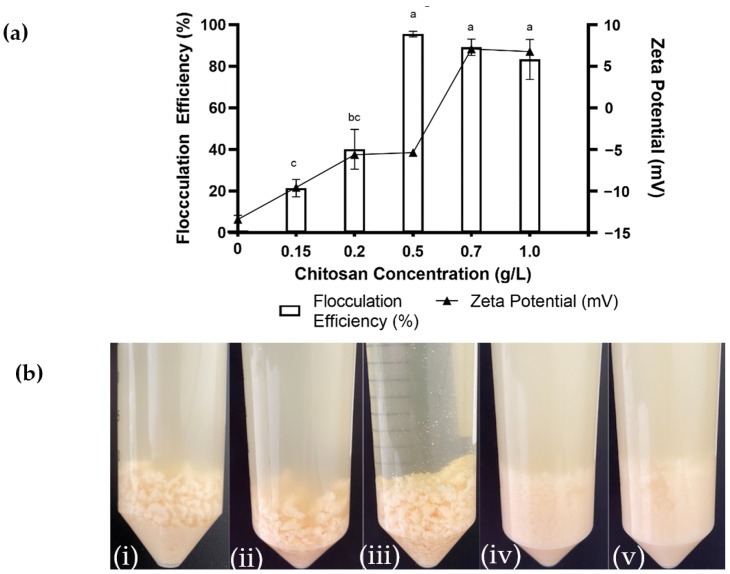
Effect of various chitosan concentrations (g/L) on *Aurantiochytrium* sp. SW1 flocculation. (**a**) Flocculation efficiency (%) and zeta potential value of remaining free cells in supernatant. (**b**) Sequence of flocs formation after flocculation at different concentrations of medium-molecular-weight chitosan: (**i**) 1 g/L; (**ii**) 0.7 g/L: (**iii**) 0.5 g/L; (**iv**) 0.2 g/L, and (**v**) 0.15 g/L. Value and error bars represent the mean and standard deviation from three replicate measurements (*n* = 3). a–c means with different letters in the same figure legend are significantly different at *p* ≤ 0.05.

The effect of the chitosan concentration on flocculation efficiency on *Aurantiochytrium* sp. SW1 cells was investigated by subjecting a 120 h SW1 culture to different concentrations of medium-molecular-weight chitosan (190–310 kDA) at the final pH of 6.6. Figure 1 shows the flocculation efficiency of SW1 cells using different concentrations of medium-molecular-weight chitosan.

The highest flocculation efficiency (95.58%) was achieved at a chitosan concentration of 0.5 g/L but decreased to 83.36% as it increased to 1 g/L. The flocculation efficiency dropped sharply with a decrease in chitosan concentration to 33.33% and 18.48% when more diluted chitosan concentrations were used (0.2 and 0.15 g/L, respectively). This shows that the chitosan concentration affects cell flocculation efficiency, as reported in previous studies [26,31]. It can be seen that a high chitosan concentration can still form large visible flocs but is ineffective because the supernatant was still cloudy, whereas at optimal concentrations (0.5 g/L), the supernatant was clear (Figure 1), which is consistent with the flocculation efficiency. Conversely, at low chitosan concentrations, the flocs formed are fine and hardly visible. The lower harvesting efficiencies observed at the higher concentrations of chitosan were due to the excessive chitosan causing the restabilization of cells that then repel each other and reduce the frequency of flocs being formed [32]. However, at a low chitosan concentration, the cell charges cannot be completely neutralized by chitosan, causing cells in a stable state to spread throughout the medium. Previous microalgae flocculation studies using chitosan show that the chitosan flocculation capacity (g chitosan/g biomass) achieved was among the most efficient (0.03 g/g) compared to previous reports on other photosynthetic freshwater microalgae cultures, *Chlorella vulgaris* and *Nannochloropsis salina* (Table 1). This is likely due to different species having different surface charge values, which will affect chitosan’s charge neutralization efficiency.

To further validate the flocculation efficiency, the value of zeta potential was correlated with the flocculation parameters tested (Figure 1). Flocculation usually occurs when the zeta potential value is near zero due to charge neutralization between the flocculant and cells [35]. At high chitosan concentrations (1 g/L and 0.75 g/L), the zeta potential values of the remaining free cells in the supernatant were 6.79 mV and 7.1 mV, respectively. This was due to the excess chitosan causing charge inversion in the cells, re-stabilization, and thus contributing to the positive zeta potential values. The zeta potential increased to −5.36 mV when the cultures were treated with 0.5 g/L, resulting in a 95.58% flocculation efficiency, in comparison to the controls (−15 mV). The increasing zeta potential value nearing zero indicates efficient neutralization occurred at this optimal chitosan concentration. Conversely, when a low chitosan concentration was used (0.15 g/L), significantly lower flocculation efficiency was observed. This is due to the insufficient chitosan to optimally neutralize cell surface charges, which was proven by the highly negative zeta potential value of the remaining free cells.

### 2.2. Effect of Molecular Weight

**Figure 2:**
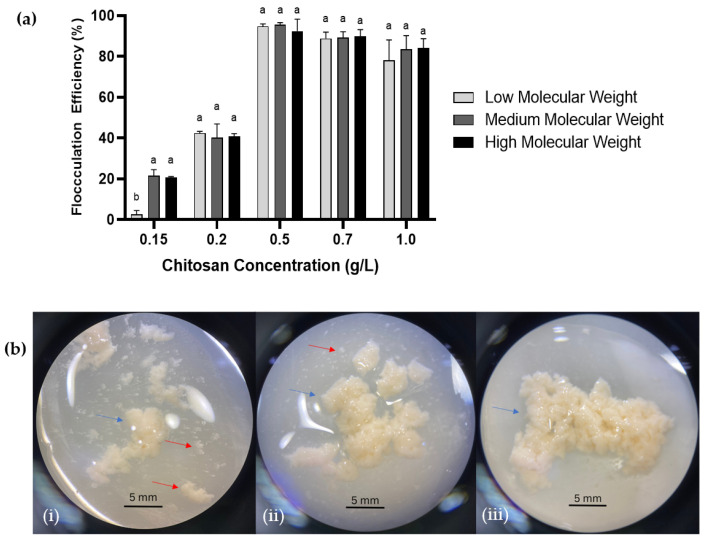
Effect of chitosan molecular weight and concentration on *Aurantiochytrium* sp. SW1 flocculation. (**a**) Flocculation efficiency and (**b**) physical appearance of flocs magnified using a dissecting microscope at different molecular weight chitosan: (**i**) Low molecular weight; (**ii**) medium molecular weight, and (**iii**) high molecular weight. Blue arrows indicate formation of macroflocs whereas red arrows indicate microflocs (Scale bar = 5 mm). Value and error bars represent the mean and standard deviation from three replicate measurements (*n* = 3), respectively. a,b means with different alphabets in the same figure legend are significantly different at *p* ≤ 0.05.

The experiments were repeated using high- (310–375 kDA) and low-molecular-weight (50–190 kDA) chitosan using the same concentration range at pH 6.6. Both showed the highest flocculation efficiency of >90% at 0.5 g/L, similar to the optimal concentration achieved with medium-molecular-weight chitosan (Figure 2). Therefore, different chitosan molecular weights showed no significant impact on the flocculation of SW1 cells. This is similar to what was reported by Low and Lau [36] in which the effect of the chitosan molecular weight becomes less significant at the optimum concentration.

However, the morphology of the formed flocs showed observable differences where rigid and more compact flocs formed with the application of high and medium-molecular-weight chitosan whereas looser and fragile flocs formed from flocculation using low-molecular-weight chitosan (Figure 2). This could possibly be caused by the bridging of microflocs due to the longer chains of high molecular chitosan, resulting in the physical associations between the microflocs, which eventually leads to the formation of macroflocs [37]. Conversely, low-molecular-weight chitosan with a shorter polymer chain results in inefficient bridging, hence generating smaller-sized flocs. Therefore, medium-molecular-weight chitosan at a 0.5 g/L concentration was selected for subsequent experiments.

### 2.3. Effect of pH

**Figure 3:**
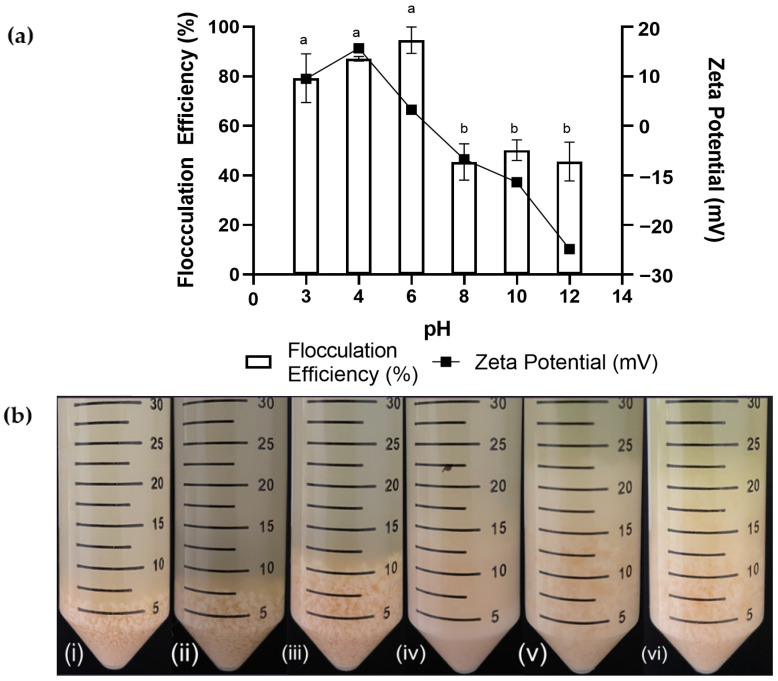
Effect of pH on *Aurantiochytrium* SW1 flocculation. (**a**) Flocculation efficiency (%) and Zeta potential value of remaining free cells. (**b**) Sequence of flocs formation after flocculation at different pH: (**i**) pH 3; (**ii**) pH 4; (**iii**) pH 6; (**iv**) pH 8; (**v**) pH 10, and (**vi**) pH 12. Value and error bars represent the mean and standard deviation from three replicate measurements (*n* = 3), respectively. a,b means with different letters in the same figure legend are significantly different at *p* ≤ 0.05.

Experiments were carried out using a 120 h culture of SW1 with chitosan (0.5 g/L) at pH 3, 4, 6, 8, 10, and 12. pH was adjusted via the addition of either acetic acid (1 M) or NaOH (1 M) to achieve the desired pH during flocculation. The results showed that when the pH of the culture was reduced from 8.53 (the initial pH of the culture) to pH 6, the flocculation efficiency reached the maximum level (94.63%) (Figure 3). The flocculation efficiency decreased up to 79.32% as the pH further decreased to pH 3. On the other hand, a rapid drop in the flocculation efficiency was also observed when the pH increased from 6 to 8. The flocculation efficiency remained between 40 and 47% as the pH further increased to 12. At the optimal pH (pH 6), larger flocs with well-defined edges were formed compared to the lower pH, which resulted in smaller-sized flocs (Figure 3). This study shows that pH significantly affects flocs’ formation and appearance. Furthermore, as flocculation was performed at a high pH (>pH 8), fine-sized flocs, which were easily broken, formed.

This shows that pH significantly affects chitosan flocculation capability, as reported previously [38]. The decreasing flocculation efficiency observed as the pH was reduced to 3 was likely due to the protonated amine group. This was evidently shown by the high positive zeta potential value at low pH 3 and 4 (9.51 and 15.7 mV, respectively) (Figure 3). Although chitosan is positively charged at a low pH due to the protonation of amine groups, excessive positive charges can cause a repulsion force between destabilized flocs, thus lowering flocculation efficiencies as pH decreases [39]. Conversely, at higher pH, chitosan started to lose its charge due to the deprotonation of the amine group on the polymeric chain. Additionally, a study conducted by Jusoh et al. [40] and Hao et al. [41] on *Chlorella vulgaris* and *Anabaena variabilis* cultures, respectively, also reported a similar increase in the negativity of the zeta potential values as pH increased. This causes chitosan to be unable to neutralize the cell’s surface charges effectively, as shown by the increasingly negative zeta potential value of up to −24.85 mV at pH 12. At this point, flocculation shows a better harvesting efficiency (96.43%) compared to centrifugation (84.28%) at 12,000× *g* for 10 min. Harvesting *Aurantiochytrium* sp. SW1 cells via centrifugation is inefficient due to their energy-intensive and ineffective nature. According to Figure S1, not all cells become pellets, as a fraction of vegetative cells containing lipids still remain at the top or are diffused in the supernatant after centrifugation, which was also reported by other researchers [17,18,19].

However, previous studies claimed that chitosan is more effective for marine microalgae (*Nannochloropsis* sp) flocculation at high pH (>pH 7.5), where it precipitated [21]. In contrast to the findings of those reported data, *Aurantiochytrium* sp. SW1′s optimal pH for flocculation by chitosan was at a slightly acidic pH (pH 6). *Nannochloropsis* and *Aurantiochytrium* are both marine microbes and the level of salt concentration for their culture media is different, where *Nannochloropsis* requires >25 g/L of salt while *Aurantiochytrium* sp. SW1 requires only 6 g/L. It has been shown that salt concentration does not affect the chitosan flocculation efficiency. A study conducted by Garzon-Sanabria et al. [42] shows that the salt concentration does not affect chitosan flocculation capabilities when tested in a low salt concentration (5 g/L) and high salt concentration (35 g/L) medium. Therefore, this difference could likely be caused by the differences in the cell surface between different species, which will affect the flocculation parameters of chitosan.

### 2.4. Culture Age and Cell Density

**Figure 4:**
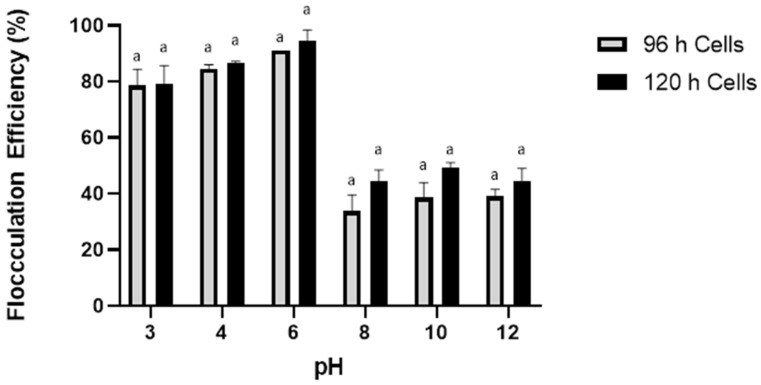
Effect of culture age on *Aurantiochytrium* sp. SW1 flocculation. Value and error bars represent the mean and standard deviation from three replicate measurements (*n* = 3), respectively. a means with different letters in the same figure legend are significantly different at *p* ≤ 0.05.

The effect of culture age and cell density on *Aurantiochytrium* sp. SW1 by chitosan was investigated using SW1 cultures cultivated for 96 and 120 h using 0.5 g/L chitosan at pH 6. Both culture ages represent the late stationary and lipid accumulation phases where most thraustochytrids cultures are harvested. As shown in Figure 4, no statistical differences in flocculation efficiency between the two culture ages were observed when flocculation was carried out at various pH (pH 3 to pH 6).

As an oleaginous microorganism, *Aurantiochytrium* sp. SW1 begins to accumulate lipids in the excess carbon source after the depletion of nitrogen at 48 h until 120 h, where a high lipid content was observed at 96 h (6.7 g/L) and started to drop due to lipid turnover at 120 h [43]. Lipid turnover was caused by the depletion of all available carbon sources where oleaginous microorganisms use reserved lipids for growth and biomass production through the β-oxidations process. This indicates that the progression of the cultures from 96 to 120 h, although resulting in significantly different lipid content, does not alter other cell properties that would affect flocculation mechanisms by chitosan. This is parallel to the observations by Vu et al. [44] where a less pronounced difference in the harvesting efficiency of *Chlorella vulgaris* was observed when flocculation using a cationic polyacrylamide polymer (FO3801) was performed on cultures in the late exponential and stationary phase. However, an approximately two-fold decrease in the harvesting efficiency was observed when flocculation was performed on early exponential cultures. This could likely be due to the significant difference in cell surface properties that occurs during the growth phase and fewer changes occurring as the cells matured and entered the stationary phase.

**Figure 5:**
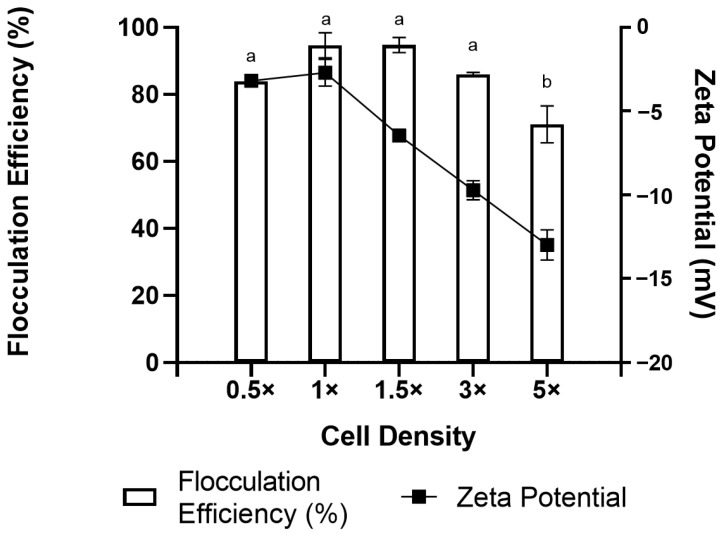
Effect of cell density on flocculation efficiency and zeta potential of *Aurantiochytrium* sp. SW1 flocculation. Value and error bars represent the mean and standard deviation from three replicate measurements (*n* = 3), respectively. a,b means with different letters in the same figure legend are significantly different at *p* ≤ 0.05.

When experiments were carried out with different cell densities (0.5×, 1×, 1.5×, 3×, and 5×) of the initial biomass concentration (15 g/L), a decreasing trend of flocculation efficiency was observed with increasing cell density (Figure 5). The optimal flocculation efficiency was achieved when the cell density was increased up to 1.5× of the initial culture and fell to <90% when the cell density of the cultures was 3× or higher. This is reflected by the zeta potential profiles where it was near zero below a 1.5× cell density and dropped to −14.1 mV sharply as the density increased. Although flocculation still occurred, the drop in the zeta potential value indicating the available chitosan (0.5 g/L) is insufficient to neutralize most of the available cells in the media as the density increases. The data obtained will help in the flocculation process during upscaling and high-cell-density cultures in the future.

## 3. Materials and Methods

### 3.1. Aurantiochytrium sp. SW1 Cultivation

Seawater Nutrient Agar (SNA), which comprises 28 g/L of nutrient agar and 18 g/L of sea salt, was used to maintain the *Aurantiochytrium* sp. SW1 culture. Stock culture from slant agar was streaked on the SNA plate to obtain single colonies. A strip of agar containing approximately ten single colonies was transferred to a 300 mL seed culture medium (in 500 mL flask) containing 60 g/L of fructose (sterilized and added separately), 2 g/L of yeast extract, 8 g/L of monosodium glutamate (MSG), and 6 g/L of sea salt. This seed culture was incubated in an incubator shaker at 30 °C and 200 rpm for 48 h. Then, a 10% *v/v* seed culture inoculum was inoculated to 2.7 L production media containing the same medium composition as the seed culture medium. The cultures were incubated in a benchtop bioreactor for 96 and 120 h at 30 °C and 500 rpm [43].

### 3.2. Flocculation Experiment

#### 3.2.1. Flocculant Preparation

The bio-flocculant used in this study was dry chitosan powder with three different molecular weights (low molecular weight, medium molecular weight, and high molecular weight) and was purchased from Sigma Aldrich (Darmstadt, Germany). A stock of 5 g/L was prepared by dissolving 1 g chitosan in 200 mL of 1% acetic acid solution under continuous agitation using a magnetic stirrer at 500 rpm for 2 h until most of the chitosan dissolved. This solution was diluted further with deionized water until the desired concentration was obtained.

#### 3.2.2. Flocculation Optimization for Harvesting *Aurantiochytrium* sp. SW1

Flocculant optimization was performed via the one-factor-at-a-time (OFAT) approach to investigate the effect of the chitosan concentration and pH level on flocculation efficiency and zeta potential. Briefly, 40 mL of the culture sample was added to 5, 3.5, 2.5, 1, or 0.75 g/L of chitosan (for all molecular weights) to achieve the final flocculant concentration of 1, 0.7, 0.5, 0.2, or 0.15 g/L, respectively. The mixture was mixed thoroughly using a magnetic stirrer at high speed (500 rpm) for 1 min and was mixed slowly (100 rpm) for the next 5 min. Then, the mixture was gently poured into a 50 mL centrifuged tube to allow the flocs to settle for 20 min via gravitational sedimentation. Afterward, the supernatant was taken approximately 2 cm from the surface for the determination of optical density (OD) at 660 nm using a spectrophotometer. The flocculation efficiency (%) was obtained using the formula shown in Equation (1)

Flocculation efficiency (%) = (OD_0_ − OD_i_)/(OD_0_) × 100% (1)

where OD_0_ and OD_i_ are defined as OD values of the initial culture without chitosan treatment (negative control) and the supernatant after flocculation, respectively [45].

After the optimal chitosan concentration and molecular weight were obtained, the next pH optimization experiment was then performed. Different aged cultures of *Aurantiochytrium* sp. SW1 (96 and 120 h) were used to further investigate the effect of culture age on flocculation efficiency. The final culture pH was adjusted to pH 3, 4, 6, 8, 10, and 12 after the addition of the flocculant. Then, the flocculation experiment proceeded as mentioned previously. Finally, to investigate the upscaling potential, the flocculation experiment was performed on different cell density cultures using the optimal parameter obtained previously.

### 3.3. Zeta Potential Analysis

Zeta potential (𝜁) was measured using the Malvern Zetasizer Nano-ZS (Worcester, United Kingdom, model ZEN3500). The supernatant was collected at a depth of 2 cm below the surface after flocs had been settled for 20 min in order to determine the zeta potential of the remaining free cells. In addition, the *Aurantiochytrium* sp. SW1 culture was also sampled for zeta potential measurements to compare the degree of charge neutralization after flocculation.

### 3.4. Statistical Analysis

An analysis of variance (One-way ANOVA) was performed followed by Tukey’s multiple comparison test to determine the significance of the difference between flocculation efficiency using Graphpad Prism 9 (GraphPad Software, San Diego, CA, USA). The significance level was set at *p* < 0.05, all experiments were performed in triplicate (*n* = 3), and the values were denoted as mean ± standard deviation.

## 4. Conclusions

This experiment showed that *Aurantiochytrium* sp. SW1 can be flocculated by chitosan efficiently. The highest flocculation efficiency (>95%) was achieved using 0.5 g/L of chitosan at pH 6. Of all the parameters tested, the chitosan concentration, pH, and cell density significantly affected flocculation efficiency. This study also demonstrates the correlation between the flocculation efficiency and zeta potential measurement, proving that a high flocculation efficiency has near-zero zeta potential values. Flocculation could provide an efficient and cheap early de-watering process in conjunction with other mechanical procedures, although other operational and cost-affecting parameters need to be considered. It will become more beneficial for large-scale fermentation (e.g., 1000 L) in which a high volume of water needs to be removed. This research was performed using the one-factor-a-time (OFAT) approach. Thus, flocculation parameters can be further optimized to achieve a more cost-effective and efficient *Aurantiochytrium* sp. SW1 flocculation.

## Figures and Tables

**Table 1:** Chitosan flocculation parameters of different microbial species. Data are means of three replicates ± standard deviation.

Optimal Concentration	Flocculation Capacity (g Chitosan/g Biomass)	Species	Biomass, g/L	Flocculation Efficiencies, %	References
0.5 g/L	0.03	*Aurantiochytrium* sp. SW1	15.0 ± 2.83	95.58 ± 1.35	This study
0.25 g/L	0.2	*Chlorella vulgaris*	1.2	91.9 ± 2.6	[33]
0.05 g/L	0.1	*Chlorella vulgaris*	0.5	92.3 ± 2.5	[33]
0.05 g/g	0.05	*Nannochloropsis salina*	0.95	97	[34]

## Data Availability

Data are contained within the article or Supplementary Materials.

## Supplementary Materials

The following supporting information can be downloaded at: https://www.mdpi.com/article/10.3390/md21040251/s1. Figure S1. (A) Aurantiochytrium sp. SW1 recovery using centrifugation at different speeds for 10 min. (B) Microscopic examination on supernatant after centrifugation at 400× magnification. The supernatant was turbid because some cells were suspended in the supernatant, not because of cell rupture. Top layer and supernatant indicating there are still lipid-rich cell (containing lipid droplet).
