# Supplementary material for: Harvesting Aurantiochytrium sp. SW1 via Flocculation Using Chitosan: Effects of Flocculation Parameters on Flocculation Efficiency and Zeta Potential
Source: Mar Drugs. 2023 Apr 19;21(4):251. doi: 10.3390/md21040251 (PMC10143672; doi:10.3390/md21040251)
Supplement: Supplementary file 1 [file marinedrugs-21-00251-s001.zip › marinedrugs-2278897-supplementary.pdf]

Supplementary Data 1:

A

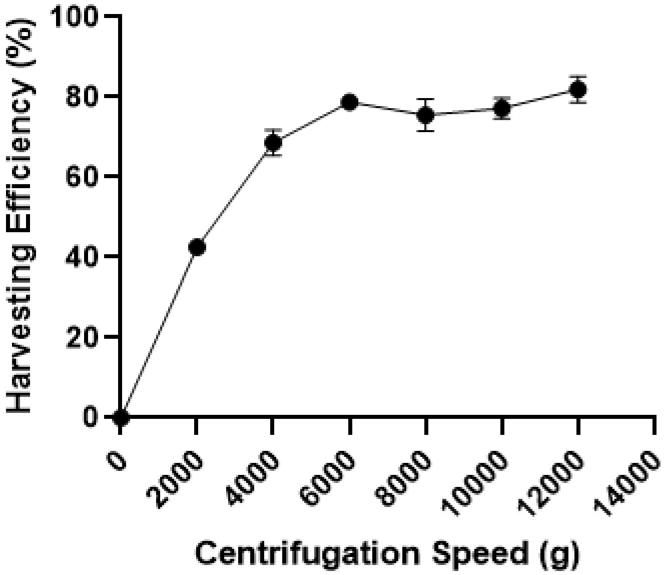

B

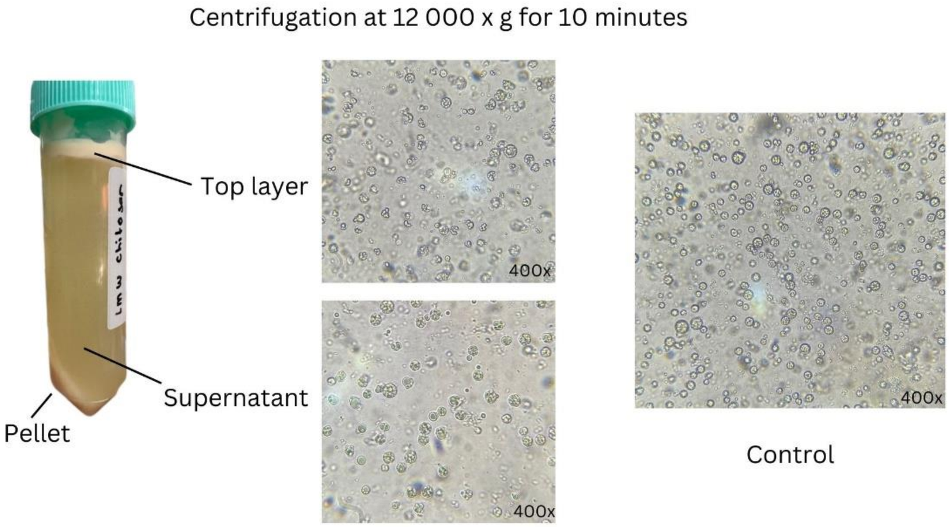

Figure S1. (**A**) *Aurantiochytrium* sp. SW1 recovery using centrifugation at different speeds for 10 minutes. (**B**) Microscopic examination on supernatant after centrifugation at 400x magnification. The supernatant was turbid because some cells were suspended in the supernatant, not because of cell rupture. Top layer and supernatant indicating there are still lipid-rich cell (containing lipid droplet).
